# Adjuvant SOX chemotherapy versus concurrent chemoradiotherapy after D2 radical resection of locally advanced esophagogastric junction (EGJ) adenocarcinoma: study protocol for a randomized phase III trial (ARTEG)

**DOI:** 10.1186/s13063-021-05617-7

**Published:** 2021-10-30

**Authors:** Jinwen Shen, Xiu Zhu, Yian Du, Yuan Zhu, Pengfei Yu, Litao Yang, Zhiyuan Xu, Ling Huang, Yunli Zhang, Yanqiang Zhang, Luying Liu, Xiangdong Cheng

**Affiliations:** 1 Department of Abdominal Radiotherapy, Cancer Hospital of the University of Chinese Academy of Sciences (Zhejiang Cancer Hospital), 1 East Banshan Road, Hangzhou, 310022 P.R. China; 2 Zhejiang Provincial Research Center for Cancer of Upper Gastrointestinal Tract, 1 East Banshan Road, Hangzhou, 310022 P.R. China; 3 Department of Pathology, Cancer Hospital of the University of Chinese Academy of Sciences (Zhejiang Cancer Hospital), 1 East Banshan Road, Hangzhou, 310022 P.R. China; 4 Department of Abdominal Surgery, Cancer Hospital of the University of Chinese Academy of Sciences (Zhejiang Cancer Hospital), 1 East Banshan Road, Hangzhou, 310022 P.R. China

**Keywords:** Esophagogastric junction adenocarcinoma, D2 radical resection, Adjuvant chemoradiotherapy

## Abstract

**Background:**

Survival benefit of adjuvant radiotherapy for locally advanced gastric cancer following gastrectomy plus D2 lymphadenectomy has always been controversial. Esophagogastric junction (EGJ) adenocarcinoma, which is usually classified as gastric cancer in East Asia, often has a higher locoregional recurrence rate after operation because of its special anatomical characteristics. The aim of this study is to determine whether adjuvant radiotherapy can improve survival of locally advanced EGJ adenocarcinoma after D2 radical resection.

**Methods:**

In this phase III, randomized, open label, controlled trial, we plan to recruit 378 patients with Siewert type II and III adenocarcinoma of EGJ, who had undergone transabdominal radical surgery and D2 lymphadenectomy, and were divided into pathological stage IIB to IIIC. All patients will be randomized 1:1 to receive either adjuvant chemotherapy alone (control group) or adjuvant chemotherapy plus chemoradiotherapy (experimental group). Patients allocated to control group will receive eight cycles of S-1 plus oxaliplatin (SOX), while the experimental group will receive two cycles of SOX followed by 45-Gy RT combined with S-1 and four additional cycles of SOX. The primary endpoint is 3-year disease-free survival rate (DFS). The secondary endpoints are 3-year overall survival rate (OS), 3-year locoregional recurrence-free survival rate (LRFS), 3-year distant metastasis-free survival rate (DMFS), and quality of life (QoL).

**Discussion:**

In the past, the adjuvant treatment of EGJ adenocarcinoma needs to draw on the experience of esophageal adenocarcinoma or gastric adenocarcinoma. In this study, EGJ adenocarcinoma is considered as an independent disease, and the conclusion will provide evidence for optimal adjuvant therapy of locally advanced EGJ adenocarcinoma after D2 radical resection.

**Trial registration:**

ClinicalTrials.govNCT03973008. Registered on 1 June 2019 (retrospectively registered), URL: https://clinicaltrials.gov/ct2/show/NCT03973008?term=NCT03973008&draw=2&rank=1

**Supplementary Information:**

The online version contains supplementary material available at 10.1186/s13063-021-05617-7.

## Background

Gastric cancer is one of the most threatening cancers, which ranked fifth in global cancer incidence and third in global mortality [1]. Surgical resection still remains the only radical treatment option for gastric cancer, while radical resection plus D2 lymph node dissection is currently the standard surgical procedure [2].

For locally advanced gastric cancer, the locoregional recurrence rate and distant metastasis rate are still high after D2 radical resection [3, 4], leading to poor prognosis of these patients. Therefore, adjuvant chemotherapy after radical surgery is currently recommended for all locally advanced gastric cancer. Pivotal CLASSIC trial, which was conducted in the East Asia, has demonstrated the superiority of adjuvant CAPOX chemotherapy in improving OS for postoperative patients with stage II/III gastric cancer [5]. Recently, ARTIST 2 trial has confirmed that adjuvant SOX can prolong DFS than S-1 monotherapy in D2-resected, node-positive gastric cancer patients [6].

However, the value of adjuvant radiotherapy after radical gastrectomy for gastric cancer has always been controversial [7]. Although the landmark INT-0116 trial confirmed the survival benefit of postoperative radiotherapy in resectable gastric cancer, most patients in this study underwent D0 or D1 resection [8]. Subsequent clinical studies have shown that adding adjuvant radiotherapy to chemotherapy does not seem to bring additional survival benefits to gastric cancer patients undergoing D2 radical resection [9].

In the past, the treatment of esophagogastric junction (EGJ) adenocarcinoma was based on the principles of esophageal cancer or gastric cancer. But it is by now increasingly recognized that EGJ adenocarcinoma is an independent tumor entity [10]. Due to the particularity of its anatomical location, the thoroughness of surgery for EGJ adenocarcinoma, especially in the aspect of surgical margin and extent of lymph node dissection [11], is not comparable to distal gastric cancer. Logically, there is a possibility of benefit from adjuvant radiotherapy for EGJ adenocarcinoma. Therefore, we initiated this prospective, randomized controlled trial to determine whether adjuvant chemotherapy plus chemoradiotherapy is superior to adjuvant chemotherapy after D2 radical resection for locally advanced EGJ adenocarcinoma, and to compare adjuvant chemotherapy plus chemoradiotherapy with adjuvant chemotherapy in terms of local recurrence, distant metastasis, and adverse reactions.

### Rationale for this trial

EGJ adenocarcinoma is a kind of tumor with high heterogeneity [12]. At present, Siewert classification is commonly used for selecting therapeutic strategies [13]. The staging and treatment strategies of type I often refer to esophageal cancer, while type II/III tumors are treated using strategies of gastric cancer, especially in East Asia. For Siewert type II/III cancer with a length of esophageal invasion ≤ 3 cm, there is a general consensus in Japan that it should be treated by an abdominal, transhiatal approach [11]. Transabdominal surgery has the advantages of thoroughness of abdominal lymph node dissection and a low rate of complications, but it also has some shortcomings such as limited proximal margin, poor surgical view of lower mediastinum, and inability to perform middle or upper thoracic lymphadenectomy.

In Europe, neoadjuvant chemoradiotherapy is the standard treatment modality for esophageal and EGJ cancers [14]. The CROSS study, which compared preoperative chemoradiotherapy plus surgery with surgery alone for esophageal and EGJ cancers, showed that the R0 resection rate was significantly increased, and the overall survival rate was also improved (49.4 months vs. 24 months) in the neoadjuvant chemoradiotherapy group [15]. However, postoperative modality is advocated by clinicians in Asian. We therefore initiated a prospective, randomized controlled study to assess the hypothesized improvement of disease-free survival for locally advanced EGJ adenocarcinoma by adding radiotherapy to adjuvant chemotherapy.

## Methods

### Study objectives

The objectives of this study are:

1. To assess whether adjuvant chemotherapy plus chemoradiotherapy is superior to adjuvant chemotherapy after D2 radical resection for locally advanced EGJ adenocarcinoma in terms of 3-year DFS.
2. To compare adjuvant chemotherapy plus chemoradiotherapy with adjuvant chemotherapy in terms of 3-year OS, 3-year LRFS, 3-year DMFS, adverse reactions, and quality of life (QoL).

### Study design

This is a prospective, randomized controlled trial to evaluate the efficacy of adjuvant radiotherapy for locally advanced EGJ adenocarcinoma after D2 resection. The trial was conducted at the Cancer Hospital of the University of Chinese Academy of Sciences (Zhejiang Cancer Hospital). We anticipated that adjuvant radiotherapy can improve the 3-year DFS by 10%, thus providing strong evidence for adjuvant therapy for locally advanced EGJ adenocarcinoma. Our study has been registered at http://clinicaltrials.gov (ID: NCT03973008).

**Fig. 1:**
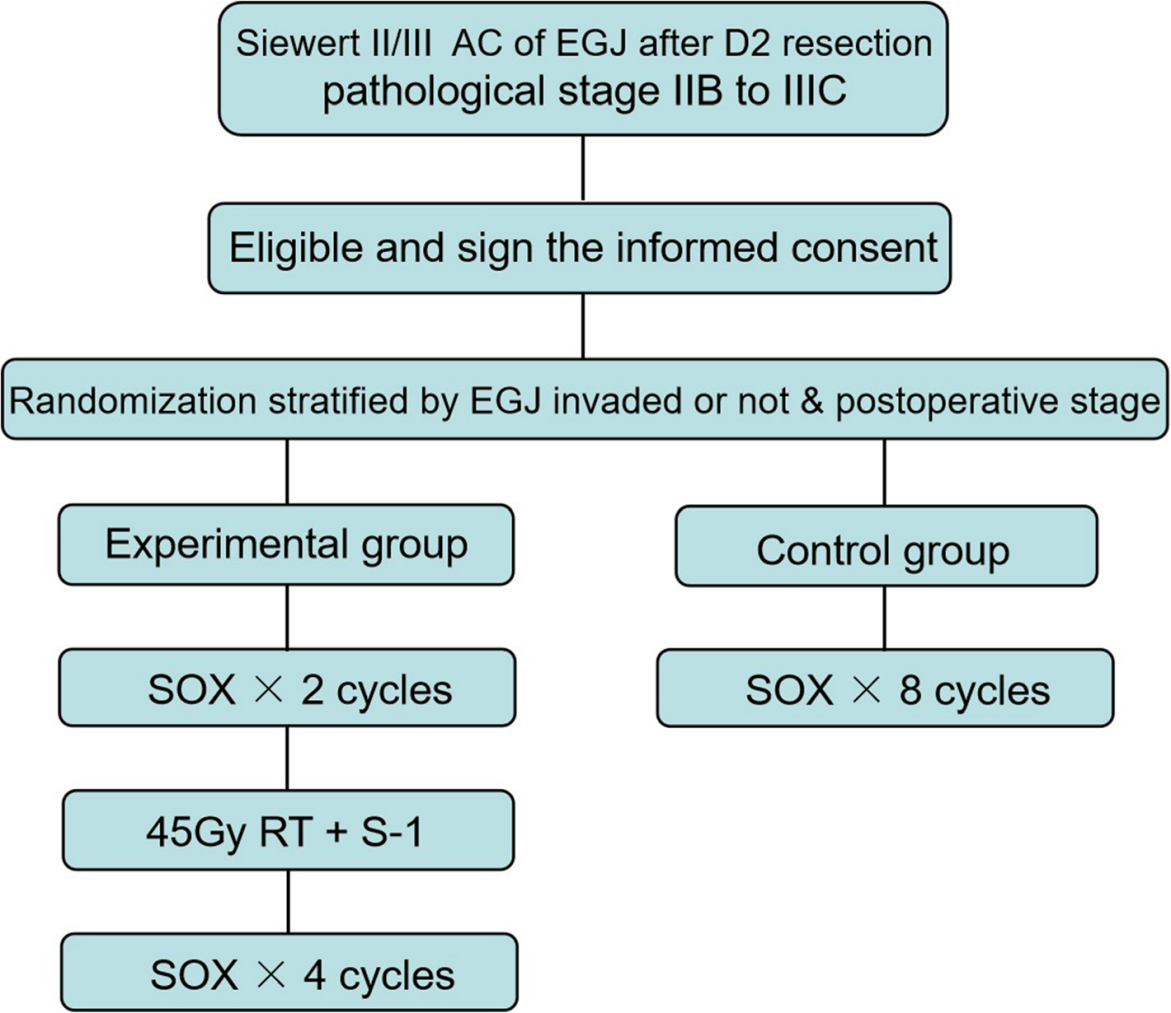
Flow chart of the study

**Fig. 2:**
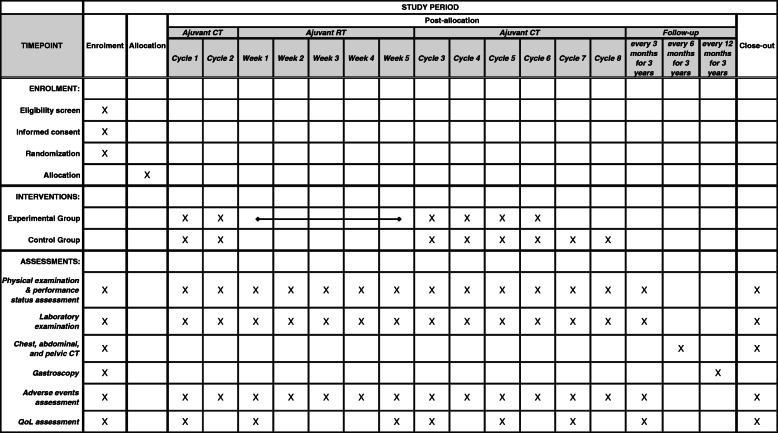
Schedule of enrollment, interventions, and assessments

After providing the signed informed consent, eligible subjects will be randomly assigned to receive adjuvant chemotherapy plus chemoradiotherapy (experimental group) or adjuvant chemotherapy alone (control group) in a ratio of 1:1. Randomization will be stratified by whether the EGJ is invaded or not and by postoperative stage. Patients in adjuvant chemotherapy group (control group) will receive eight cycles of SOX regimen chemotherapy; while patients in adjuvant chemotherapy plus chemoradiotherapy group (experimental group) received two cycles of SOX, then concurrent chemoradiotherapy (45 Gy of radiation with S-1 concurrent oral chemotherapy), followed by four additional cycles of SOX. All adjuvant therapy should begin within 4–6 weeks after surgery. Patients enrolled in this study should complete 3-year follow-up after randomization. The date and location of the first recurrence, as well as the date of death, will be documented. The study flow chart is shown in Fig. 1, and the schedule of enrollment, interventions, and assessments is shown in Fig. 2. The Standard Protocol Items: Recommendations for Interventional Trials (SPIRIT) checklist is provided in Additional file 1.

### Study population

#### Inclusion criteria

1. Voluntary participation and written informed consent.
2. Aged older than 18 years and younger than 70 years, gender unlimited.
3. Confirmed that the tumor center was located between 1 cm above and 5 cm below the EGJ, regardless of invasion to the EGJ, and histopathological diagnosis of adenocarcinoma.
4. Received transabdominal D2 radical operation, and R0 resection was performed
5. Ascites cytology was negative.
6. Postoperatively classified as pathological stages IIB~IIIC.
7. Eastern Cooperative Oncology Group (ECOG) performance status score 0–1.
8. Laboratory tests: hemoglobin (Hb) > 9 g/dL; white blood cell (WBC) > 3 × 10^9^/L; absolute neutrophil count (ANC) > 1.5 × 10^9^/L; platelet (Pt) > 100 × 10^9^/L; bilirubin < 1.5 times the upper limit of normal value; alanine-glutamic transaminase (ALT) and alanine transaminase (AST) < 2.5 times the upper limit of normal value; and serum creatinine < 1.5 times the upper limit of normal value.
9. Daily energy intake > 1500 kcal.

#### Exclusion criteria

1. Received neoadjuvant therapy.
2. Intraperitoneal implantation and distant metastasis were found.
3. History of malignant tumors (except skin basal cell carcinoma, thyroid papillary adenocarcinoma, and cervical carcinoma in situ, which survived for more than 3 years).
4. History of serious heart and lung diseases, abnormal hematological examination, and immunodeficiency.
5. Uncontrollable infections, seizures, or loss of self-awareness due to mental illness.
6. Adverse drug addiction, long-term alcoholism, and AIDS patients.
7. History of severe allergies.
8. Pregnant or breastfeeding women.
9. Participated in other clinical trials within 30 days.
10. Any other condition that researchers consider inappropriate to participate in this trial.

#### Exit criteria

1. Cannot be treated according to the scheme of the study.
2. Grade 4 allergic reactions or severe adverse reactions to research drug.
3. Disease progression in the course of the study.
4. Pregnant and/or not using adequate contraceptive measures.
5. Researchers judged that subjects should not continue the study.
6. Subjects request withdrawal.

### Chemotherapy regime

SOX regimen was used for adjuvant chemotherapy:

- Oxaliplatin 130mg/m^2^ given intravenously on day 1
- S-1 given orally twice a day (half an hour after breakfast and dinner) for 14 consecutive days, and the dose of S-1 is shown in Table 1
- Repeated every 3 weeks

**Table 1:** The dose of S-1

Patient’s body surface area (m^**2**^)	S-1 single oral dose
<1.25	40mg
≥1.25 to <1.5	50mg
≥1.5	60mg

### Concurrent chemoradiotherapy regime

- Technique of irradiation: intensity-modulated radiation therapy
- Preparation prior to CT simulation: fast 4 h and drink 100 ml of water (including 5 ml of contrast agent) 30 min prior to scan; patients with remnant stomach drink additional semiliquid diet (like congee) 10 min before scanning
- Technique of simulation: patients in the supine position with forearm on forehead, and use custom immobilization device (e.g., thermoplastic mask) to fix body from lower thorax to lower abdomen. The scanning range is from the apex of lung to the pelvic inlet, with 5-mm slice thickness. Intravenous contrast should be used whenever available unless allergic to contrast, old age, or with serious complications.
- Irradiation field: the clinical target volume (CTV) include anastomosis with a 3-cm margin in the cranio-caudal direction, and regional lymph node drainage area [No.110 (below carina), 20, 1-3, 7-9, 11 (proximal 1/3), 16a1-2]. The planning target volume (PTV) will be obtained by expanding the CTV by 5–7mm in radial direction, and by 10mm in cranio-caudal direction.
- Dose prescription and fractionation: a total dose of 45 Gy at 1.8 Gy per day, 5 days per week, for 5 weeks. Dose prescription and recording should comply with the recommendations of the ICRU 83.
- Organ at risk (OAR) volume definition and dose constraints: the complete volumes of the remnant stomach, the liver, the kidneys, the lungs, and the heart have to be delineated. Intestine and spinal cord must be outlined the volume between above and below 2cm range of PTV. In this study, the normal tissue dose constraints are shown in Table 2.
- Concurrent chemotherapy: S-1 given orally twice a day (half an hour after breakfast and dinner), Monday to Friday, during radiotherapy, and the dose of S-1 is shown in Table 1.

**Table 2:** Normal tissue dose constraints

Organs	Dose constraints
Remnant stomach	V40 < 50% (no hot spot)
Liver	V30 < 40%
	Dmean < 18Gy
Kidney	V20 < 30%
Lung	V20 < 20%
Heart	V30 < 30%
Intestine	Dmax ≤ 50Gy
Spinal cord	Dmax < 40 Gy

### Outcome measures

#### Primary endpoint

The 3-year DFS is the primary endpoint of this study. It is defined as the percentage of patients without locoregional recurrence, distant metastasis, or tumor-related death at 3 years measured from the date of randomization. Once a patient is lost after the last follow-up, his/her survival time will be censored at the last date the patient is known to be alive.

#### Secondary endpoints

The secondary endpoints of this study included 3-year OS, 3-year LRFS, 3-year DMFS, and QoL.

Three-year OS is defined as the percentage of patients in this study who are still alive at 3 years measured from the date of randomization. In this study, locoregional recurrence was defined as recurrence at the anastomotic site, tumor bed, remnant stomach, or regional lymph nodes site, and distant metastasis is defined as non-regional lymph node recurrence, peritoneal seeding, and metastasis to other organs. Three-year LRFS and DMFS are defined as the percentage of patients who are without locoregional recurrence or distant metastasis at 3 years measured from the date of randomization respectively. EORTC QLQ-C30 and QLQ-STO22 questionnaires will be used to evaluate QoL of patients at baseline, every 4 weeks during treatment and every visit after treatment.

### Safety reporting

#### Adverse events

Adverse events are defined as any adverse medical event that occurs between the inclusion in the study with the signing of the informed consent and the last visit, regardless of whether there is a causal relationship with the drugs or treatments being studied.

Adverse events include the following:

- All suspected adverse drug reactions (ADR)
- All reactions due to drug overdose, abuse, withdrawal, allergy, or toxicity
- Obviously unrelated diseases, including the aggravation of pre-existing diseases
- Injury or accident
- Abnormalities found by physiological or physical examinations and requiring clinical treatment or further examination (unlike repeated validation examinations); abnormalities found in laboratory tests require clinical treatment or further investigation (unlike repeated validation tests); if these abnormalities are related to another reported event (e.g., elevated liver enzymes in jaundice patients), they should be described in the notes to the clinical event report and not listed as a separate adverse event.

#### Adverse event record

Research physicians should use concise medical terminology to report all adverse events directly observed by physicians or spontaneously reported by subjects. In addition, patients should be asked about adverse events every time they visit a doctor at the beginning of treatment, fill in the adverse event record table truthfully, and record the occurrence time, severity, duration, measures taken and outcome of adverse events. Adverse events should be recorded in the adverse events table of case report form (CRF).

#### Grading of adverse events

Adverse events were classified into grades 1–4 according to the National Cancer Institute Common Terminology Criteria for Adverse Events version 4.0 (NCI-CTCAE v4.0) and the European Organization for Research and Treatment of Cancer (EORTC)/Radiation Therapy Oncology Group (RTOG) score:

- Grade 1: Mild, asymptomatic, or mild symptoms, only clinical diagnosis or symptoms, can be tolerated
- Grade 2: Medium, has a certain impact on normal life
- Grade 3: Severe, unable to carry out normal daily activities; leading to hospitalization or prolonged hospitalization
- Grade 4: Life threatening, if emergency intervention is needed; otherwise, there is a direct risk of death
- Grade 5: Death

#### Serious adverse events

When an adverse event meets one or more of the following criteria, it is classified as a serious adverse event (SAE):

- Causing death
- Life-threatening
- Need for hospitalization or extended hospitalization
- Causing persistent or severe disability or dysfunction
- Congenital deformity or birth defect
- Major medical events

Any serious adverse events in the course of clinical research must be reported by fax or telephone to the ethics committee and the principal investigator within 24 h. The principal investigator will collect data and reports according to SFDA and Ministry of Health’s reporting requirements on adverse reactions. Researchers must fill in a serious adverse event form to record the occurrence time, severity, duration, measures taken, and outcome of serious adverse events.

Once SAE occurs, all antineoplastic therapy should be discontinued immediately, and the relationship between adverse events and research drugs or radiotherapy should be assessed, and appropriate symptomatic and supportive treatment should be given until the patient recovers or remains stable.

### Dose modification

According to CTCAE v4.0, the toxicities during the study should be graded. When adverse drug reactions occur, the dose of drugs should be reduced according to the following principles, or even discontinue treatment. Details of each patient’s dose reduction and withdrawal should be recorded in the CRF table.

#### Dose reduction/withdrawal principle for adverse drug reactions of concurrent chemotherapy and adjuvant chemotherapy

- No special treatment should be given when grade 1 adverse reactions occur
- S-1/oxaliplatin should be discontinued and given symptomatic treatment when grade 4 leucopenia, grade 3 gastrointestinal adverse reactions, grade 2 anemia and thrombocytopenia, and grade 2 liver and kidney dysfunction occur. If the grade of adverse reactions dropped to 0–1 within 5 days after treatment, the initial dose of S-1/oxaliplatin was restored; if not, consideration should be given to reducing dose of S-1/oxaliplatin to 80% of the initial dose. If the above adverse reactions persist for more than 3 days after symptomatic treatment and dose reduction, or other adverse reactions with new grade 2 or higher occur again, S-1/oxaliplatin should be terminated.
- When other grade 3 adverse reactions occur, the treatment principle is the same as that of corresponding grade 2 adverse reactions
- If any grade 4 adverse reactions occur, the concurrent chemotherapy or adjuvant chemotherapy should be stopped and not continued.

#### Principles of interruption or discontinuation of radiotherapy

- When the acute grade 3 radiation toxicities occurs, radiotherapy should not be interrupted and symptomatic treatment should be given. If it does not recover to grade 0–1 within 7 days, radiotherapy should be interrupted and restarted after the grade of toxicity return to 0–1. If the same toxicities of above grade 2 reoccur, radiotherapy should be terminated. When radiotherapy is terminated, S-1 should be terminated at the same time.
- If any grade 4 adverse reactions except leukopenia occurred, radiotherapy should be discontinued. When the adverse reactions recover to grade 0–1, restart radiotherapy, but not chemotherapy.
- Dose of radiation should not be adjusted unless new adverse reactions occur or the original adverse reactions are aggravated.

### Follow-up

Patients should be followed up once every 3 months within 2 years after operation and once every 6 months 2 years after operation until 5 years. Follow-up contents include physical examination, performance status monitoring, weight monitoring, routine blood test, blood chemistry, tumor markers (including CEA and CA19-9), adverse events, and QoL assessment. Chest, abdominal, and pelvic CT should be performed every 6 months, and gastroscopy is recommended once a year. If new symptoms or symptoms worsen, patients should receive follow-up visit at any time. The recurrence and/or metastasis must have biopsy or clinical imaging evidence, and the time and location of recurrence and/or metastasis should be recorded in detail.

### Ancillary and post-trial care

Only when treatment-related complications occur, the corresponding ancillary care is permitted. There is no prearranged post-trial care.

### Statistical analysis

In this study, the sample size was estimated by statisticians. The 3-year DFS of the experimental group is expected to be increased from 47 to 60% (δ=0.13), which provides strong evidence for adjuvant treatment of locally advanced EGJ adenocarcinoma. With a one-sided alpha =0.05 and 1 − *β*=0.8, 172 per group would be necessary, and 206 endpoint events need to be observed. Given an estimated dropout rate of 10%, the required total sample size would be 378. Patients enrolled in this study would be randomly divided into experimental group and control group, stratified by whether the EGJ is invaded or not and by postoperative stage. The ratio of the two groups was 1:1.

The analysis was based on the intention-to-treat (ITT) population. The ITT population should include all patients receiving randomization. Per-protocol (PP) population should include all patients who received at least two cycles of chemotherapy and completed radiotherapy for experimental group and received at least four cycles of chemotherapy for control group.

Comparison of baseline data between the two groups: if the quantitative data is normal distribution, *t* test is used; if it is not, rank sum test is used; *χ*^2^ test is used for qualitative data. Survival analysis is carried out by SPSS software package. Log rank *χ*^2^ analysis is used for comparison between the two groups, and Cox regression model is used in multivariate analysis. *χ*^2^ test is used to compare the recurrence rate, metastasis rate, and the incidence of side effects between the two groups. Two-tailed test is used, and *p* < 0.05 is considered statistically significant.

### Randomization, sequence generation, and implementation

All potential participants will be initially screened by the designated pathologists and then further screened according to the inclusion criteria and exclusion criteria by the designated clinicians. Those eligible subjects who signed the consent will be randomized to either experimental group (adjuvant chemotherapy plus chemoradiotherapy) or control group (adjuvant chemotherapy alone) in a ratio of 1:1. Randomization will be stratified by whether the EGJ is invaded or not and by postoperative stage.

We adopt a mobile application software, developed by Fudan University Biostatistics Central Office (Shanghai, China), to perform randomization, using varied permuted block design with a block size 2–6. The generation of random allocation sequence is operated by Fudan University Biostatistics Central Office, and the principal investigator will be informed by text message to ensure allocation concealment. Then, the principal investigator will forward the message of treatment assignments to the designated investigators, who guarantee the participants will be assigned to the corresponding intervention/control group.

### Data collection, management, and monitoring

Clinical researchers will collect baseline information, treatment efficacy, and toxicity of enrolled patients. When the data is collected, the designated data processors will check them, hide private information, and then enter the data into an online electronic CRF, which is developed by an independent data collection group (yitu-med, https://edcs.yitu-med.com).

No external data monitoring committee will be established because the treatment in this trial has been widely used in clinic. The internal data monitoring committee will be composed of the principal investigator and senior clinicians, who will be mainly responsible for safety data review and evaluation.

### Ethics

This study was carried out according to the Declaration of Helsinki. The protocol, the informed consent form, and the case report form have been reviewed and approved by the medical ethics committee of our hospital (IRB-2019-196), and the main members who participated in the study have obtained the certificate of the Good Clinical Practice (GCP) course.

Participation in this study is completely voluntary. All patients will be informed of the study process, benefits, costs, potential adverse reactions, and other alternative treatment options in detail before entering this study, and then sign the informed consent. All the signed informed consent will be kept by the principal investigator. After entering this study, patients could withdraw from the study at any time for any reason and receive other alternative treatment.

### Auditing and protocol amendments

The GCP center of our hospital will audit the clinical trials in progress annually, including this study.

During this study, if the principal investigator considers it necessary to amend the research protocol, he shall first submit an application for protocol amendments to the ethics committee and then notify all participants after the protocol amendments are reviewed and approved by the ethics committee.

### Dissemination policy

The results of this study will be published in academic journals or academic conferences.

## Discussion

The role of adjuvant radiotherapy after radical resection for gastric cancer has always been controversial. The classic INT-0116 study [8], which showed that radiation therapy for postoperative gastric cancer patients improved 3-year DFS and OS compared with surgery alone, could not prove the benefit of adjuvant radiotherapy after radical D2-resection due to limited lymph node dissection in that study (D2 dissection accounted for only 10% ). The Korean ARTIST 1 study [9], which included patients underwent D2 lymphadenectomy, indicated that the addition of radiotherapy to adjuvant chemotherapy did not significantly reduce the recurrent rate after surgery. Subgroup analysis suggested that pathologic node-positive patients might benefit from postoperative irradiation. But no difference in 3-year DFS was found for patients with lymph node metastasis after D2-resection when adding irradiation to SOX chemotherapy, according to the results of ARTIST 2 study [6].

However, it is worth noting that the majority of patients enrolled in trials on gastric cancer in Asia were distal gastric cancer, while the proximal gastric cancer was fewer. For example, cancer of the gastric antrum and gastric body accounted for 60% of the patients in ARTIST 1 study [9], while proximal gastric cancer accounted for only 20%. Due to the particularity of anatomical location, the benefit of postoperative radiotherapy for EGJ cancer cannot be directly analogized by the conclusions of studies on gastric cancer.

Adenocarcinoma of the esophagogastric junction have the characteristic of high heterogeneity. Siewert classification, which is a classification method based on the location of tumors, is being widely used in guiding the clinical treatment of EGJ adenocarcinoma. On the view of surgical approach, Siewert type I tumor is usually treated by transthoracic approach, while type II/III tumors have two ways of surgery: transthoracic approach and transabdominal approach. A phase III trial in Japan recruited patients with Siewert type II/III EGJ adenocarcinoma, whom were randomly divided into the left thoracoabdominal approach group (LTA) and the transhiatal approach group (TH). The interim analysis showed that the risk of death in LTA group was 36% higher than that in TH group [16], so the trial was closed ahead of schedule and concluded that for type II/III tumors, the left thoracoabdominal surgery was inferior to the transhiatal surgery. Recently, a meta-analysis of 8 studies, involving 1155 patients with EGJ cancer who underwent transthoracic surgery or transhiatal surgery, found a shorter hospital stay, lower 30-day hospital mortality, and decreased pulmonary complications in the transhiatal group. Because of a potential survival advantage for type III tumors in the transhiatal group, the authors recommended the transhiatal approach as the optimal choice, especially for Siewert type III tumors [17].

However, both transabdominal and transthoracic operations often have the shortcomings of limited proximal or distal margin, incomplete dissection of mediastinal or abdominal lymph nodes, which make the local recurrence rate of EGJ cancer unsatisfactory. So, multimodal therapy has become the standard treatment strategy for locally advanced EGJ tumors. The MAGIC study in the UK, which compared the efficacy of perioperative chemotherapy with surgery alone for resectable gastroesophageal cancer, showed that the 5-year survival rate of the perioperative chemotherapy group increased by 13% compared to that of surgery alone group (5-year OS rate, 36% vs. 23%) [18]. The France FNCLCC/FFCD study is similar to the MAGIC study, but included more EGJ cancer (account for 60%), and the survival rate of the chemotherapy-treated patients increased by 14% (5-year OS rate, 38% vs. 24%) [19]. In Asia, however, clinicians and patients prefer postoperative therapeutic modality for three reasons: first, the preoperative clinical stage is not as accurate as the postoperative pathological stage, which leads to over treatment of some early patients; second, the toxicity caused by neoadjuvant radiotherapy may preclude surgery or increase post-operation complication [20]; and third, the frequency of distal intramural spread in irradiated tumor is increased, which results in the difficulty of safety margin determination [21]. Therefore, we designed this phase 3, randomized controlled study to provide an optimal adjuvant treatment strategy for locally advanced EGJ cancer.

## Trial status

The protocol version number and date: Version 2.1, 29 November 2019

The date of recruitment start: 1 July 2019

The expected date of recruitment complete: 30 June 2022

## Supplementary Information

**Additional file 1.** Standard Protocol Items: Recommendations for Interventional Trials (SPIRIT) checklist.

## Data Availability

Not applicable.

## Abbreviations

ADR: Adverse drug reactions
ALT: Alanine-glutamic transaminase
ANC: Absolute neutrophil count
AST: Alanine transaminase
CRF: Case report form
CTV: Clinical target volume
DFS: Disease-free survival rate
DMFS: Distant metastasis-free survival rate
ECOG: Eastern Cooperative Oncology Group
EORTC: European Organization for Research and Treatment of Cancer
GCP: Good clinical practice
Hb: Hemoglobin
ITT: Intention-to-treat
LRFS: Locoregional recurrence-free survival rate
OAR: Organ at risk
OS: Overall survival rate
Pt: Platelet
PTV: Planning target volume
QoL: Quality of life
RTOG: Radiation Therapy Oncology Group
SAE: Serious adverse event
SOX: S-1 plus oxaliplatin
WBC: White blood cell
